# A qualitative exploration of critical issues limiting local vaccines production in Nigeria: lessons from the COVID-19 pandemic

**DOI:** 10.1186/s12889-025-24949-6

**Published:** 2025-12-30

**Authors:** Obi Peter Adigwe, Godspower Onavbavba, Diana Oyin-mieyebi Wilson, Olajide Joseph Adebola, Anthony Ayeke, Kenneth Anene Agu

**Affiliations:** 1 National Institute for Pharmaceutical Research and Development, Plot 942, Cadastral Zone C16, Idu Industrial District, Abuja, Federal Capital Territory Nigeria; 2 Home Plus Medicare Services Ltd. F1, Police Cooperative Estate, Obot Akara Street, Beside Army Estate Kubwa, Abuja, Federal Capital Territory Nigeria; 3 European Union Delegation to the Federal Republic of Nigeria and ECOWAS, Abuja, Federal Capital Territory Nigeria; 4 Howard University Global Institute Nigeria, Abuja, Federal Capital Territory Nigeria

**Keywords:** Qualitative, Manufacturing, Vaccines, Public health, Prevention, Medicines’ security

## Abstract

**Background:**

Vaccines are essential health interventions that have become a cost-effective tool for maintaining public health. Access to vaccines in Nigeria and other developing countries is, however, affected by various relevant issues, including insufficient supply, inequitable distribution, vaccine hesitancy, and a lack of local manufacturing capacity. This study, therefore, aimed to gain an in-depth understanding of the challenges impeding vaccine production in Nigeria, taking lessons from the COVID-19 pandemic.

**Method:**

A qualitative study was undertaken using a purposive sampling strategy to recruit key stakeholders across Nigeria’s vaccine value chain. Interviews were carried out using a semi-structured interview guide. Relevant stakeholders with varying levels of experience across the vaccine value chain were interviewed to ensure that robust and comprehensive data were obtained. The data collected were subjected to thematic analysis, following the interpretivist tradition. Throughout the study, trustworthiness was assured by adopting relevant processes such as data triangulation, reflexive and audit trails. Ethical approval was obtained from the National Institute for Pharmaceutical Research and Development Health Research Ethics Committee prior to the commencement of data collection.

**Results:**

This study revealed eight thematic areas underpinning the gaps associated with local vaccine production in Nigeria. Challenges included limited capacity, inadequate research and development, infrastructure deficits, and a sub-optimal environment for vaccine development. Furthermore, the challenges associated with commercialisation and distribution were identified. Findings also revealed a lack of critical factors required for expediting vaccine manufacturing in Nigeria, including poor commitment to collaboration, an absence of sustainability policies and sub-optimal legislation.

**Conclusion:**

The study provides insight into systemic and structural barriers to local vaccine production in Nigeria. It identifies key areas requiring urgent intervention, which include funding, infrastructure, human capacity, and regulatory reform. Addressing these gaps through multi-level strategies is critical for improving vaccine self-sufficiency. Evidence from this study can help develop contextual strategies that will expedite vaccine production and Medicines’ Security in Nigeria.

**Supplementary Information:**

The online version contains supplementary material available at 10.1186/s12889-025-24949-6.

## Background

Vaccines are essential public health commodities that confer individual and herd immunity against infectious diseases [1]. They represent a cost-effective and fundamental strategy for maintaining public health [2]. The availability of vaccines has been reported to provide significant public health improvements, evidenced by the global declines in morbidity, mortality, and the cost of treatment against communicable tropical diseases. According to global health reports, vaccination prevents two to three million deaths annually [3]. This was further reiterated during the COVID-19 pandemic, wherein vaccines were reported to have reduced the number of hospitalisations [4, 5]. Further assessment revealed that the efficacy of immunisations in protecting against COVID-19-related mortality was about 63%, preventing 19.8 million deaths in 185 countries and territories during the first year of COVID-19 vaccination [6].

However, in low- and middle-income countries, inadequate access to vaccines limited the potential impact of this intervention, especially in terms of protection against mortality during the pandemic [7]. Across Africa, the late arrival of COVID-19 vaccines, compounded by issues such as limited supply [7] and logistical challenges, contributed to the continent having the lowest vaccination coverage globally, with only 31.1% of its population fully vaccinated against COVID-19 as of September 2023 [8]. In Nigeria, evidence suggests that 77 million of the 116 million eligible persons have completed the primary series of vaccination, and less than a quarter of this population has received a booster dose since the process commenced in the country [9, 10]. These disparities in vaccination coverage were, among other factors, driven by insufficient supply, hesitancy, inequitable distribution, and limited vaccine production in Africa [11].

According to the World Health Organisation, there is a limited production of vaccines across Africa [6]. The continent produces just about 1% of its total vaccine needs, despite accounting for almost a fifth of the world’s population [12]. This is equivalent to fewer than 100 million doses for a population of over 1.4 billion [13]. For many countries across the continent, this dearth in vaccine production is supplemented by supplies from donor agencies such as UNICEF and the Global Vaccine Alliance (GAVI). However, during the COVID-19 pandemic, the inequity and delays in vaccine supply highlighted the gaps associated with reliance on vaccine imports. In Nigeria, total dependence on the importation of vaccines significantly exacerbated the nation’s Medicines’ Security challenges during the pandemic [14]. Therefore, to promote sustainable public health solutions, it is necessary for governments to expedite actions to ensure vaccine production in the country.

It is, however, unclear why vaccine manufacturing in Nigeria has remained limited, despite the country’s history of successful mass vaccine production against smallpox, measles, and yellow fever [15]. In 2020, the COVID-19 pandemic also stimulated dialogues and initiatives towards revitalising local production of vaccines in the country [16], yet significant progress has not been made. The review of the extant literature reveals that there is a paucity of information regarding this, as only a few quantitative studies were identified to have been undertaken to proffer an understanding of the bottlenecks associated with a lack of access to vaccines in the country [14, 17]. The study, therefore, aims to provide an in-depth understanding of the challenges regarding vaccine production by generating novel insights into the phenomena that are difficult to investigate quantitatively. Understanding these challenges from the perspectives of relevant stakeholders across the vaccine value chain in Nigeria can guide policies and practices towards expediting the local production of vaccines against routine and emergent infectious diseases.

## Methods

### Study setting and design

The Study was conducted in Nigeria. The conceptual framework by Creswell [18] was adopted to proffer an understanding of the proposed methodology that best describes the phenomena to be studied. Whilst vaccines have been identified as a key public health intervention, the gaps associated with their lack of production in Nigeria are yet to be robustly explored and comprehensively articulated. Following the review of relevant literature, the adoption of a qualitative methodology was crucial to explore the views of stakeholders in the vaccine value chain in a manner that provides novel insights as well as meets all the critical functions of science [19]. Therefore, an exploratory qualitative research approach was selected to guide the interpretive generation of the challenges associated with local vaccines’ production in this study.

### Sampling

A purposive sampling method was employed to recruit participants who were knowledgeable about the phenomenon. The selected stakeholders represented different professional backgrounds and areas of practice across the vaccine value chain. Each respondent was therefore recruited based on their diversity in years of experience, professional backgrounds, and variance in practice settings. Participants were eligible for inclusion if they had at least ten years of work experience in vaccine-related fields such as pharmaceutical manufacturing, regulatory affairs, public health policy, or immunization logistics. They were further identified based on their professional roles, involvement in national or institutional decision-making processes, and prior contributions to vaccine production and related policy development. This ensured the recruitment of individuals capable of providing rich, reflexive insights grounded in both practice and policy. The sampling strategy, therefore, ensured that themes generated from the analysis were robust enough to yield insight into the phenomenon. Participants were invited using emails detailing the purpose of the study.

Inclusion criteria for selected samples in the study were stakeholders who had been practising in the vaccine value chain for a minimum of ten years and were willing to participate. Practitioners who did not meet these criteria were not included in the study, meaning that the cohort comprised only those with the requisite experience and abilities enabling the provision of critical information. Following Creswell’s [18] guideline for qualitative research, about 18 to 30 participants were appropriate for inclusion in the study, to ensure that non-hypothetical modes of qualitative research are achieved. However, saturation of data was the critical determinant of the sample size used in the study.

### Data collection

Data were obtained through semi-structured interviews. The choice of an interview method was influenced by the necessity to explore information in a manner that allowed participants to express themselves without influence [19]. The data collection tool was a well-designed interview guide that comprised open-ended questions structured to obtain demographic information and to explore general ideas and reflexive perspectives on the gaps associated with vaccine production in the country. Before the commencement of data collection, the interview guide was pretested with a small group of participants who shared similar characteristics with the target population. This pretesting phase helped assess the clarity, relevance, and sequencing of the questions. The interviews were conducted physically and through Zoom, and participants were allowed to choose the method they preferred. For each participant, interviews were conducted for a period of 30 to 40 min. The interviews unfolded such that respondents critically explored issues they felt were essential to the subject matter without influencing their anonymity. The interview sessions were recorded using the Zoom recorder and an external digital recording device. Consent for recording was obtained from participants before the interview. Saturation was achieved upon the 16th interview when no new theme was derived. However, two sessions were further undertaken, which affirmed saturation at the 18th interview, given that emergent themes only buttressed previously developed themes. All interviews were conducted in English and subsequently transcribed verbatim and encrypted for confidentiality.

### Data analysis

The transcribed interviews were allotted identification numbers to maintain the anonymity of each participant. Transcriptions were then analysed using thematic analysis [20]. This iterative process involved individual and group-level review and interpretation of the narrative data. First, the entire transcript was read through to ensure familiarity with the dataset, after which three levels of coding were done. The first level of coding involved a line-by-line de novo scanning of transcripts to obtain emerging ideas. Constant comparison of the emergent codes was conducted consistently to ascertain similarities and peculiarity in words or statements that were unique to the study. Codes were further developed into code structures representing a brief contextual definition or properties of codes. These code structures were eventually clustered into sub-themes. Memo drafts were generated afterwards to compare and narratively provide textual descriptions in a manner that explains and facilitates understanding of the phenomenon represented by each sub-theme. Third-level coding was then achieved by synthesising all sub-themes as a composite structure to yield the emergent themes. Each theme was structured to address dominant areas of interest informed by the perspectives of stakeholders as challenging the production of vaccines in Nigeria.

### Quality in data management

Rigour was ensured in this study through the adoption of recommended validation techniques [21, 22]. Verbatim transcripts, field notes, memos, reflexive journals, and the subjectivity statements of the researchers were constantly referred to, throughout the various phases of the study. This was done to eliminate bias and clarify the researchers’ position. The objectivity and reliability of the study were further ensured via an audit trail. This involved the review and confirmation of the analysis of data by an independent reviewer. Furthermore, internal and external validity in this study was demonstrated using rich descriptions in answering questions presented on the Critical Appraisal Skills Programme (CASP) checklist [22]. The questions appraising the checklist were suitably answered in a manner that strongly affirmed the study's credibility.

## Results

### Demography

Table 1 provides an overview of the socio-demographic characteristics of 18 stakeholders who participated in the study. The demography represents 1 policymaker, 2 health administrators, 3 regulators, 3 healthcare professionals, 3 manufacturers, and 6 researchers with varying levels of experience that spanned 17 to 40 years in practice. The findings record that 9 of the respondents practised in the public sector, about 6 participants were private sector players, and the others practised in development agencies. More male participants compared to female counterparts were recruited in the study. Stakeholders with a doctoral degree emerged as the highest proportion, in terms of the educational qualifications considered in the study.

**Table 1:** Socio-demographic characteristics of the study participants

Participant number	Gender	Role in the vaccine value chain	Years of practice	Highest level of qualification	Sector of practice
Respondent 1	Male	Health administrator	23	Fellow, WAPCP	Public
Respondent 2	Male	Researcher	26	Doctoral degree	Private
Respondent 3	Male	Researcher	18	Doctoral degree	Public
Respondent 4	Male	Healthcare professional	35	Doctoral degree	Private
Respondent 5	Male	Researcher	30	Doctoral degree	Public
Respondent6	Male	Manufacturer	20	First degree	Private
Respondent 7	Male	Health Administrator	23	Master’s degree	Public
Respondent 8	Male	Healthcare professional	32	First degree	Development agency
Respondent 9	Male	Regulator	20	First degree	Public
Respondent 10	Male	Policy maker	38	Doctoral degree	Public
Respondent 11	Male	Manufacturer	40	Master’s degree	Private
Respondent 12	Female	Manufacturer	19	First degree	Private
Respondent 13	Male	Researcher	17	Doctoral degree	Private
Respondent 14	Male	Regulator	20	Master’s degree	Development agency
Respondent 15	Male	Regulator	33	Doctoral degree	Development agency
Respondent 16	Male	Healthcare professional	18	Doctoral degree	Public
Respondent 17	Male	Researcher	26	Doctoral degree	Public
Respondent 18	Male	Researcher	32	Doctoral degree	Public

*WAPCP* West African Postgraduate College of Pharmacists

### Findings

**Fig. 1:**
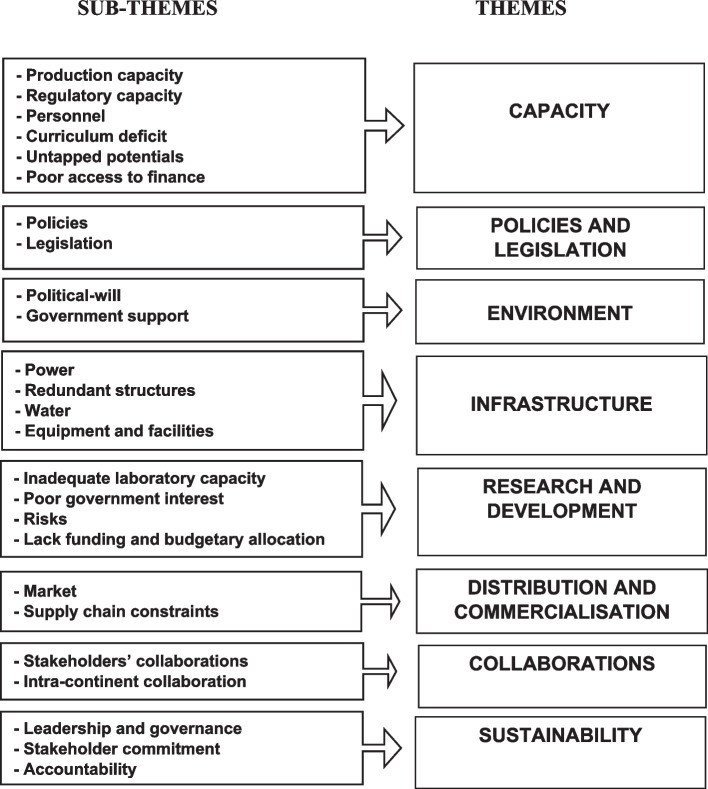
Flow diagram showing themes and sub-themes of findings

Eight major themes emerged from the data analysis, which characterised participants’ perceptions of the challenges associated with the lack of vaccine manufacturing in Nigeria. The themes were classified into policies and legislations; challenges with the environment, which included political will and government support; as well as capacity gaps, which comprised sub-themes highlighting production and regulatory capacity, personnel, curriculum deficits, poor access to finance, and untapped potential. Figure 1 provides a summary of the various themes and sub-themes that emerged from the analysis of data.

1. Capacity

Respondents indicated capacity deficits as a critical factor challenging the production of vaccines in Nigeria. The participants primarily described this as a form of expertise. Consequently, six factors were highlighted in this category as constraints in capacity development towards vaccine manufacturing. The first is the technical expertise to produce.

a Production capacity

Stakeholders highlighted that the nation’s complete dependence on vaccine imports has served as a major disincentive to developing capacity towards vaccine production. In a remark by a pharmaceutical manufacturer,

“…*a lot of them are not WHO certified…we do not produce raw materials, and all the excipients and APIs are imported into this country, we are disadvantaged*… (Respondent 11, male, with a Master’s degree and 40 years of experience in pharmaceutical manufacturing)

In a similar light, another manufacturer indicated that the country’s deficit in the production of sterile preparations also challenges its capacity to venture into the manufacture of vaccines.

“…*when it comes to sterile products, we are not there. If we had facilities that manufacture sterile products, we would have built capacity in Nigeria that can eventually migrate into the production of vaccines*…” (Respondent 9, male, with a first degree and 20 years of experience in regulatory affairs).

b Regulatory capacity

As highlighted, manufacturing vaccines requires higher technicalities and regulations than the production of other medical products. Findings from the study revealed that there exist deficits in regulation towards vaccine production in the country. Participants stated that currently*,*

*“…there are no regulations and guidelines for vaccines in Nigeria…*” (Respondent 9, male, with a first degree and 20 years of experience in regulatory affairs).

“…*there is a weak understanding and implementation of GMP for vaccine production…*” (Respondent 5, male, with a doctoral degree and 30 years of experience in research and development**)**

Whilst the National Agency for Food and Drug Administration and Control (NAFDAC) recently attained Maturity Level 3 (ML3) for drug regulation in the country, a respondent in the regulatory sector affirmed that,

*“…ML3 does not speak to vaccine manufacture… the regulator must achieve the lot release and also achieve level 4 for you to produce vaccines that will be acceptable internationally*… (Respondent 14, male, with a master’s degree and 20 years of experience in regulatory affairs)

iii. Personnel

Stakeholders further indicated that there is limited human resource capacity for vaccine production in the country*.* According to researchers in the vaccine value chain,

“…*there is a shortage of personnel, and we have to define means where we increase the personnel…”* (Respondent 13, male, with a doctoral degree and 17 years of experience in research and development).

“…*vaccine production has failed in Nigeria because of poorly trained manpower…”* (Respondent 5, male, with a doctoral degree and 30 years of experience in research and development).

This factor eventually led to poor personnel confidence in venturing into vaccine development in the country.

*“…a reasonable number of persons are not confident in themselves that they can go into vaccine production*…” (Respondent 10, male, with a doctoral degree and 38 years of experience in polic*y* making).

iv. Curriculum deficit

Further insights into the gaps in personnel training were traced to deficits in the curriculum of Universities. For this reason, a policymaker highlighted that some personnel may lack understanding of the requirements for vaccine production.

“…*look at the Nigerian University curriculum; no University is doing vaccinology*…” (Respondent 13, doctoral degree, male, with 17 years of experience in research and development).

*“…six out of ten pharmacists may not be able to understand what vaccine production and distribution is all about*…” (Respondent 10, male, with a doctoral degree and 38 years of experience in policy making).

e Untapped potentials

Whilst pharmacists are critical professionals who are trained to be drug experts, stakeholders indicated that these professionals, alongside other practitioners who have been trained in vaccinology outside of Nigeria, find it difficult to apply theoretical knowledge into practice.

*“…we go out of the country to do the training, we return to Nigeria, and there is nowhere to put it into practice*…” (Respondent 3, male, with a doctoral degree and 18 years of practice in research and development).

According to the stakeholders**,** the human and technical potentials for vaccine production in Nigeria have not been effectively harnessed, and they opined that it will be challenging to go into vaccine production with the current situation.

*“*…*we have not looked inward to see*…” (Respondent 1, male, a fellow of WAPCP with 23 years of experience in health administration).

*“…most of the research going on in research institutes is ending up on the shelves and in the library… we have really not been able to industrialise the outputs from our research*…” (Respondent 5, male, with a doctoral degree and 30 years of experience in research and development).

*“…most of the research institutes and Universities are not really being utilised…* (Respondent 18, male, with a doctoral degree and 32 years of experience in research and development).

f Poor access to finance

The participants expressed the difficulties associated with accessing funds for local pharmaceutical manufacturing, highlighting that vaccine production was capital-intensive and that secondary barriers to funding limit capacity development in this area.

“… *for any local investor, finance is a very significant challenge*…” (Respondent 6, male, with a first degree and 20 years of experience in pharmaceutical manufacturing).

*“…accessing funds is impossible. You can’t even get it…we couldn’t access the funds because the banks had other criteria that had nothing to do with what we wanted to do… the banks do not have the same requirements to access the funds*…” (Respondent 6, male, with a first-degree and 20 years of experience in pharmaceutical manufacturing).

2. Policies and legislation

a Policies

The study findings highlighted policies and legislation as critical and interrelated factors affecting vaccine production in the country.

*“…the legislation and policies on import, and clearing of products are not favourable for manufacturers*…” (Respondent 12, female, with 19 years of experience in pharmaceutical manufacturing).

b Legislation

Some of the respondents stated that the policies in Nigeria were robust and unanimously alluded to legislative inadequacies as a critical challenge associated with the development of vaccines in the country.

*“…the policies are robust, and Nigeria has never been lacking in policies*…” (Respondent 16, male, with a doctoral degree and 18 years of experience as a healthcare professional).

*“…we don’t have proper legislation that will support that kind of investment in our system today…”* (Respondent 4, male, with a doctoral degree and 35 years of experience as a healthcare professional).

3. Environment

The deficits in pharma related legislation also contributed to perceived adequacies of an ecosystem with the potential to catalyse vaccine production in the country. The respondents highlighted two critical areas impeding the provision of an enabling environment*.*

a Political-will

Participants indicated that, without the political will of the Government, vaccine production in the country could not be achieved.

*“…the issue of political will, because it is only when a government determines to achieve something that they will achieve it*…” (Respondent 5, male, with a doctoral degree and 30 years of experience in research and development).

b Government support

Further insights into the gaps in political will revealed that government support was lacking. The participants identified political intrigues as a main contributor to inadequate and sub-optimal progress in the sector. According to a manufacturer, the lack of requisite sector-specific support for vaccine production has limited advancements in this area.

*“…everything is politicised. Since COVID, there has been a lot of talk about vaccine manufacturing in this country, yet we have not made any headway*…” (Respondent 11, male, with a master’s degree and 40 years of experience in pharmaceutical manufacturing).

“…*most industries pay permit levies, which is a very serious problem*…”* (*Respondent 18, male, with a doctoral degree and 32 years of experience in research and development).

“…*the credit facilities are not necessarily there, and if they were, with the current exchange rate, it becomes economically difficult for any person to invest in such a project*…” (Respondent 10, male, with a doctoral degree and 38 years of experience in policymaking).

4. Infrastructure

The existence of relevant infrastructure is a prerequisite to vaccine manufacturing. Findings from this study indicated that in Nigeria, the necessary infrastructural base for vaccine production was lacking. There emerged four critical areas of infrastructural deficits in the country.

a Power

 

The erratic supply of electricity in the country was indicated as a significant infrastructural challenge to vaccine manufacturing.

*“…as it stands now in Nigeria, we cannot generate the power that will meet the teeming population of the country, and we need this.”* (Respondent 7, male, with a master’s degree and 23 years of experience in health administration).

Participants stated that if investments were to be made currently towards vaccine production in the country,

*“…power failure can jeopardise everything*…” (Respondent 16, male, with a doctoral degree and 18 years of experience as a healthcare professional).

b Redundant structures

Stakeholders in this study indicated that there is a plethora of structures in the country that are not harnessed to facilitate vaccine development.

“…*you will be amazed at the infrastructure we have around and how redundant they are…*” (Respondent 2, male, with a doctoral degree and 26 years of experience in research and development).

“.…*we do not have our facilities replaced with modern facilities to meet modern challenges in vaccine production*…” (Respondent 5, male, with a doctoral degree and 30 years of experience in research and development).

iii. Water

Inadequacy in water supply was further identified as a constraint towards vaccine development.

“…*Adequate portable pipe-borne water is lacking*…” (Respondent 5, male, with a doctoral degree and 30 years of experience in research and development).

iv. Equipment and facilities

 

Major gaps associated with vaccine manufacturing, as highlighted from the interviews, were equipment and facilities. It was also indicated that whilst some of the required equipment for vaccine development was available, they were insufficient to meet vaccine production.

*“…there are challenges with getting the necessary materials and even fine equipment needed*…” (Respondent 10, male, with a doctoral degree and 38 years of experience in policy making).

*“…there are no facilities for people …”* (Respondent 9, male, with a first degree and 20 years of experience in regulatory affairs).

“…Some equipment that we need is not here…” (Respondent 17, male, with a doctoral degree and 26 years of experience in research and development).

5. Research and development

 

Robust production of vaccines begins at the level of research and development. However, from the respondents' perspectives, four major subthemes highlighted the challenges associated with research and development for vaccines in Nigeria.

a Inadequate laboratory capacity

Participants indicated that the research laboratories in the country had not been standardised to enable vaccine research and development.

“…*the environment in our laboratory is not yet up to the standard of vaccine production, because we need a very sterile environment, which what we have now cannot guarantee*…” (Respondent 17, male, with a doctoral degree and 26 years of experience in research and development).

“…*due to the state of our lab, we are not really deep into that yet, because there are different categories of labs that should be used for the production*….” (Respondent 18, male, with a doctoral degree and 32 years of experience in research and development).

b Poor government interest

Factors associated with the inadequate capacity for research and development in the country were also primarily attributed to poor government interest. As such, commencing vaccine production in Nigeria might be very challenging.

“…*government not being interested in research and development*…” (Respondent 1, male, fellow of WAPCP, with 23 years of experience in health administration).

“…*the country has not prioritised research as a way of developing a country*…” (Respondent 5, male, with a doctoral degree and 30 years of experience in research and development).

iii. Risks

The study further indicated that there were associated risks with vaccine research, which have constituted a significant limitation to investments in vaccine development.

“…*there are risks attached to it*…” (Respondent 1, male, a fellow of WAPCP with 23 years of experience in health administration).

*“…there are ups and downs, sometimes it may not go the way it is planned, and funds may be lost…*” (Respondent 12, female, with a first-degree and 19 years of experience in pharmaceutical manufacturing).

iv. Lack of funding and budgetary allocation

Stakeholders indicated inadequate funding for vaccine research and development in the country. Researchers in this cohort expressed the lack of budgetary allocation for R&D in the country and the unavailability of grants to support efforts towards vaccine development.

“…*financial resources dedicated to research are absent in all our budgets*…” (Respondent 4, male, with a doctoral degree and 35 years of experience as a healthcare professional).

*“…We do all these from our personal funds; there are no research grants to support*…” (Respondent 3, male, with a doctoral degree and 18 years of practice in research and development)**.**

6. Distribution and commercialisation

 

A recurring theme in this study representing a major challenge towards vaccine production in Nigeria involves the distribution and commercialisation of this public health tool. Sub-themes on market and supply chain constraints were highlighted under this category.

a Market

Despite high investment costs, the uncertainty of a favourable vaccine market has been accorded a significant constraint towards vaccine production. This has therefore constituted gaps towards vaccine investment and manufacturing in Nigeria.

“…*there have been companies that had attained WHO prequalification in the past. When it elapsed, they could not renew because the market was not forthcoming*…” (Respondent 16, male, with a doctoral degree and 18 years of experience as a healthcare professional).

“…*it is uncertain whether it is easy for businessmen to make those investments without being assured of patronage*…” (Respondent 11, male, with a Master’s degree and 40 years of experience in pharmaceutical manufacturing).

b Supply chain constraints

Another major vaccine production constraint associated with distribution and commercialisation is the supply chain management challenge. The inadequacy of storage facilities and cold chain equipment has impeded readiness towards vaccine development in Nigeria.

“…*availability is affected by the storage facility and the availability of mobility to hard-to-reach areas…*” (Respondent 4, male, with a doctoral degree and 35 years of experience as a healthcare professional).

“…*sometimes, the cold chain has been broken…the efficacy of the vaccine is now in doubt*…” (Respondent 10, male, with a doctoral degree and 38 years of experience in policy making).

The inadequacy of proper storage facilities constitutes another major challenge that can compromise vaccine integrity. These factors were considered a major concern for vaccine production in Nigeria.

“…*do we have enough storage facilities? I will say no*…” (Respondent 9, male, with a first-degree and 20 years of experience in regulatory affairs).

“…*some of the primary health centres are in areas that do not have electricity or fridges…*” (Respondent 5, male, with a doctoral degree and 30 years of experience in research and development).

7. Collaborations

The participants highlighted poor collaboration between stakeholders and amongst countries across Africa as a significant challenge constraining vaccine manufacturing.

a Stakeholders’ collaboration

Amongst stakeholders, it has been highlighted that synergy towards facilitating vaccine production is lacking.

“…*there is the politics of professional tribalism, nepotism and sectionalism…*” (Respondent 5, male, with a doctoral degree and 30 years of experience in research and development).

“…*if I want to manufacture medicines, or go into diagnostics or vaccines, they will be saying that I don’t know anything, I have no experience*…” (Respondent 6, male, with a first-degree and 20 years of experience in pharmaceutical manufacturing).

b Intra-continent collaboration

Another critical area of concern associated with the lack of vaccine production in Nigeria was the poor synergy among African countries. The issue of poor continent recognition amongst African member countries was strongly highlighted.

“…*a major problem in Africa is the issue of local recognition or mutual reliance*. *Other African countries find it difficult to accept what is coming out from the country…*” (Respondent 9, male, with a first degree and 20 years of experience in regulatory affairs).

*“…when the issue of COVID came up, there was also a vaccine produced in South Africa, but due to the African mentality, people were only thinking of what to get from the Western world, it killed their own effort*…” (Respondent 17, male, with a doctoral degree and 26 years of experience in research and development).

8. Sustainability

Participants indicated the lack of sustainability measures as a critical deterrent to vaccine manufacturing in Nigeria. Three sub-themes were therefore identified to provide a perspective into this area.

a Leadership and governance

The non-continuation of relevant initiatives following the change of leadership in government has been highlighted to contribute to the current state of vaccine development.

“…*will this have a continuity, or will it die after he/she leaves?*” (Respondent 2, male, with a doctoral degree and 26 years of experience in research and development).

“…*changes can come anytime in the government. Our main problem in Africa is leadership…”* (Respondent 7, male, with a master’s degree and 23 years of experience in health administration).

b Stakeholder’s commitment

Participants stated that one of the critical challenges towards vaccine production in Nigeria was the issue of commitment. Poor commitment of stakeholders towards achieving Medicines' Security, was indicated as a discouraging factor.

“…o*ur drive is very poor…*” (Respondent 3, male, with a doctoral degree and 18 years of practice in research and development).

“…*there is one fundamental problem—getting the right team. Getting somebody interested in the vision, not just the money*…” (Respondent 13, male with a doctoral degree and 17 years of experience in research and development).

It therefore emerged that if vision-driven stakeholders are not employed to strategically commit to the objective of local vaccine manufacturing in the country, this goal will be unattainable.

“…*no amount of policy and infrastructure can deal with human innuendos…*” (Respondent 2, male, with a doctoral degree and 26 years of experience in research and development).

iii. Accountability

Further to poor stakeholder commitment towards vaccine production in Nigeria, a lack of mutual accountability by relevant players was indicated to have contributed to the current state of local production of vaccines in Nigeria. Two priority themes that emerged were, mismanagement and corruption:

“…*there is mismanagement…*” (Respondent 5, with a doctoral degree and 30 years of experience in research and development).

“…*Nigerians, we have this lackadaisical attitude and not finishing things to the end. And also, putting corruption into so many things…*” (Respondent 7, male, with a master’s degree and 23 years of experience in health administration).

## Discussion

Findings from this study revealed that despite Nigeria being a nation with over a hundred pharmaceutical manufacturing companies [23], production of vaccines in the country is still limited by technical and human resource capacity deficits. The study revealed a significant dearth of vaccine development capacity across educational institutions, research laboratories, and the manufacturing space. This implies the lack of expertise across all levels of vaccine development, ranging from upstream to fill-finish mechanisms [24]. It further reflects the fact that many manufacturing firms are ill-equipped to produce WHO-certified sterile medicinal preparations, especially as regard capacities related to skilled personnel.

Similar challenges have been successfully addressed in other African settings. For example, Senegal’s Institut Pasteur de Dakar has leveraged strategic investments and international partnerships to produce WHO-prequalified yellow fever vaccines and develop COVID-19 diagnostics. In South Africa, the Biovac Institute moved from basic packaging to advanced fill-and-finish processes through technology transfers and a public–private ownership model [25]. Egypt’s VACSERA and Tunisia’s Institut Pasteur Tunis have each strengthened their local vaccine portfolios by combining specialized institutional capacity with collaborations involving global manufacturers [26]. These case studies demonstrate that political commitment, regulatory strengthening, and well-structured public–private partnerships can create sustainable local vaccine-manufacturing ecosystems.

Back in Nigeria, only about four local pharmaceutical manufacturers have obtained the WHO Good Manufacturing Practices (GMP) certification [27], yet none of their products have been pre-qualified. This is a deterrent to the overarching objective of expediting the production of internationally approved vaccines in the country. This further identifies the need to strengthen the drug regulatory system in Nigeria. Whilst the national regulatory agencies have secured WHO Maturity Level 3 (ML3) for vaccine licensure, findings from the study identified that the level of certification attained was insufficient for the regulation of locally produced vaccines. Although ML3 indicates a functioning regulatory system, countries must attain the Lot release as well as ML4 to become eligible to supervise and control the production of vaccines that will be acceptable internationally [28]. Therefore, capacity development through technology transfer, curriculum reforms, personnel training, and strengthening of the existing manufacturing and regulatory frameworks is critical towards the local production of vaccines.

To emphasise efficient capacity utilisation towards vaccine development in the country, an enabling environment created by the Federal Government is required. This study, however, revealed an inadequate enabling environment for the production of vaccines in the country. The local pharmaceutical manufacturers have been significantly limited by the paucity of government support, credit facilities, and incentives to drive vaccine production. This finding aligns with reports by Ugbam and Okoro [29] and therefore affirms the constraints of access to finance and foreign exchange to procure inputs for the local manufacture of this public health tool. Vaccine development is a complex and capital-intensive venture [30], and the limited access to finance hindered the ability of the Nigerian pharmaceutical sector to scale up production in this critical area. In vaccine manufacturing countries such as India and China, this challenge is being mitigated by several public financing mechanisms, as well as attrition and consolidation strategies amongst existing pharmaceutical manufacturers [31]. However, in Nigeria, the study findings report that these opportunities are lacking, and pharmaceutical industries are tasked to self-supply utilities such as power and water, which can account for as high as 40% of the overall production cost in some cases. Due to the high manufacturing expenditure, finished products manufactured locally are often unable to compete in price with imported alternatives.

The challenges bordering infrastructural deficits in Nigeria were further highlighted in terms of sub-optimal or redundant facilities. This finding validates previous studies that highlighted infrastructural inadequacy as a major factor impeding vaccine development and accessibility [32]. In terms of research and development capacity, a significant infrastructural and funding gap exists. Participants indicated that the budgetary allocation for vaccine research and development in the country was inadequate. This is a critical challenge to vaccine development, especially since sufficient investment in vaccine research is critical towards achieving sustainable production [33].

The existing risks associated with vaccine research and commercialisation were highlighted as constraints to developing this intervention in Nigeria. There are uncertain outcomes regarding the marketing of pharmaceutical products in the country. This was attributed to a lack of patronage or market guarantees. The findings identified distribution challenges and disruptions of the vaccine cold chain due to technical and infrastructural inefficiencies across the nation. This consolidates previous reports [34], representing a deviation from the National Drug Distribution Guidelines [35].

The study further revealed a lack of synergy amongst stakeholders in the vaccine value chain, thereby mirroring previous findings [36]. Regulatory organisations such as the Pharmacy Council of Nigeria (PCN), can advocate for policies that support vaccine production, develop technical guidelines and training to address human resource gaps, and facilitate public–private partnerships and international collaborations to enhance local capacity [37]. Similarly, the inadequacy of the partnership between Nigeria and other African nations was identified. Nigeria could partner more closely with multilateral agencies, leveraging Gavi’s financing instruments and pooled procurement, WHO’s technology-transfer hubs, and CEPI’s rapid-response platforms to secure the funding, technical assistance, and market guarantees needed for scale-up. Complementing these partnerships with innovative approaches such as vaccine bonds, shared manufacturing clusters, and South–South cooperation with successful producers like Institut Pasteur de Dakar and Biovac will help build a resilient, locally driven vaccine-manufacturing ecosystem. By learning from regional success stories and embracing these collaborative and innovative strategies, Nigeria can chart a pragmatic pathway toward sustainable vaccine production.

### Strengths and limitations

This study applied a robust qualitative framework, allowing for an in-depth exploration of challenges associated with the local production of vaccines in Nigeria. The purposive inclusion of stakeholders with diverse experiences across the vaccine value chain enhanced the richness of the data. Methodological rigor was ensured through pretested interview guides, and the use of audit trails to strengthen trustworthiness.

Nonetheless, certain limitations exist. The purposive sampling approach, though appropriate for qualitative inquiry, may have limited the diversity of perspectives, as stakeholders outside the researchers’ networks may have been inadvertently excluded. In addition, there was a notable gender imbalance among participants, with only one female included. Although selection was based on relevance to the study objectives and not on gender, this under-representation may, however, imply a loss of diversity in voices and limit women’s contribution in this area. Future studies may consider examining whether gender influences professional perspectives within similar contexts.

## Conclusion

Findings from this study revealed eight critical challenges associated with local vaccines’ manufacturing in Nigeria. The challenges in the areas of research and development, inadequate infrastructure, as well as distribution and commercialisation of pharmaceutical products, were highlighted. An introduction of comprehensive mechanisms that can enhance funding and infrastructural development was identified as critical to achieving local production of vaccines in Nigeria.

Human factors related to the shortage of skilled personnel, collaborations, and accountability were also identified as constituting barriers to development in the sector. Measures towards actualising strong collaboration and commitment are therefore recommended. Similarly, capacity development through technology transfer and personnel training can be utilised to address the overarching need for skilled personnel in vaccine production.

It also emerged that there is a critical need for improvement in the enabling environment that would consequently facilitate vaccines development in the country. Therefore, strengthening legislative and regulatory capacities can expedite sustainable vaccine manufacturing in Nigeria. Although this study provided novel insights regarding challenges surrounding the local production of vaccines in Nigeria and other developing countries, further research is recommended to deepen these emergent findings.

## Supplementary Information

Supplementary Material 1.

## Data Availability

The datasets used and/or analysed during the current study are available from the corresponding author on reasonable request.

## Abbreviations

CASP: Critical Appraisal Skills Programme
COVID: Coronavirus Disease
GAVI: Global Vaccine Alliance.
GMP: Good Manufacturing Practices
ML3: Maturity Level 3
NAFDAC: National Agency for Food and Drug Administration Control
R&D: Research and Development
UNICEF: United Nations Children's Fund
WAPCP: West African Postgraduate College of Pharmacists
WHO: World Health Organization
CEPI: Coalition for Epidemic Preparedness Innovations
