# Supplementary material for: A qualitative exploration of critical issues limiting local vaccines production in Nigeria: lessons from the COVID-19 pandemic
Source: BMC Public Health. 2025 Dec 30;25:4363. doi: 10.1186/s12889-025-24949-6 (PMC12754854; doi:10.1186/s12889-025-24949-6)
Supplement: Supplementary file 1 — Supplementary Material 1. [file 12889_2025_24949_MOESM1_ESM.docx]

**Appendix 1: Interview Guide**

**Perception regarding the state of vaccine provision in Nigeria**

What can you say about the current state of vaccine provision and distribution in Nigeria?

**Prompts**

- What are your perspectives on the current state of vaccines availability, and accessibility in Nigeria?
- What opportunities/advantages are currently available in Nigeria to expedite local vaccine production

**Gaps in Local Manufacturing of Vaccines in Nigeria**

Why do you think Nigeria has not been able to produce vaccines locally?

**Prompts**

What possible factors can undermine the commencement local vaccine manufacturing?

- What are your views regarding vaccines R&D and supply chain management?
- What are your opinions on the adequacy of the national vaccine policy in promoting local vaccine manufacturing?
- What is the feasibility of domestic vaccines manufacturing in Nigeria in terms of legislature and funding?

**Benefits and Implications of Local Production of Vaccines**

What should be expected from local vaccine manufacturing in Nigeria?

**Prompts**

- How will vaccine production in Nigeria affect healthcare delivery and economic advancement in the country?
- In what way will these benefits extend to Africa and the global market?
- In your opinion, what are the implications for safety and affordability with respect to local vaccine production?

**Strategies to Improve Local Production of Vaccines in Nigeria**

What measures do you feel should be adopted to improve the domestic manufacturing of vaccines in Nigeria?

**Prompts**

- How can Nigeria address the potential negative implications of local vaccine manufacturing?
- What are your views on the necessary infrastructures, technical capacity and collaborations needed to facilitate local vaccine production and access?
- What pharmaceutical industry initiatives could be implemented to foster local vaccine production?
- What are your views on the necessary legislative frameworks to expedite national vaccine sufficiency given the timeline for the new co-financing arrangement between the federal government and the vaccine alliance (GAVI)?
